# Characteristics and smoking behaviors among patients with drug-resistant tuberculosis (DR-TB) in South Africa

**DOI:** 10.1186/s12889-025-22565-y

**Published:** 2025-04-10

**Authors:** Phindile Zifikile Shangase, Brandon S. Shaw, Ina Shaw

**Affiliations:** 1 Division of Public Health, University of the Free State, Bloemfontein, South Africa; 2 School of Sport, Rehabilitation and Exercise Sciences, University of Essex, Wivenhoe Park, Colchester, Essex UK

**Keywords:** Risk factors, Smoking, Tobacco smoking, Tuberculosis

## Abstract

**Introduction:**

Cigarette smoking is an independent risk factor for drug-resistant tuberculosis (DR-TB), highlighting the importance of developing effective smoking cessation strategies tailored to specific contextual insights.The aim of this study was to assess the smoking behaviours, cessation attempts, and associated factors among patients with drug-resistant tuberculosis (DR-TB).

**Methods:**

A cross-sectional study was conducted in three specialized DR-TB public hospitals in the KwaZulu-Natal province, South Africa. Data were collected using a structured interviewer-administered questionnaire adapted from the Global Adult Tobacco Survey. 196 participants (172 males, 24 females) with an average age of 36.13 years ± 10.27 SD were included.

**Results:**

The study found 172 of the 196 participants to be male. The mean age of the participants was 36.13 years ± 10.27 SD, with 182 between the ages 21 and 50 years old. 64% had completed secondary level of education, followed by 21% who had primary schooling. 63% were unemployed. Of the participants, 95.3% smoked daily: 36.2% within five minutes of waking, 25.5% within 30 min, and 31.1% within one hour. Manufactured cigarettes were used by 84.8%, while 21.8% preferred self-rolled cigarettes. Only 64% disclosed their smoking behaviour upon hospital admission. In the past year, the following reasons were given for attempts to quit smoking, TB diagnosis (111/196), health concerns (44/196), and personal factors (20/196).

**Conclusion:**

The findings underscore the urgent need for targeted smoking cessation interventions integrated into DR-TB care, emphasizing consistent counselling, improved disclosure of smoking behaviors, and enhanced education on smoking risks to support patients in quitting.

## Introduction

While *Mycobacterium tuberculosis* (TB) is a preventable and treatable disease, in 2023, it likely regained its position as the leading cause of death globally from a single infectious agent [1]. Although the World Health Organization (WHO) African region achieved significant progress with a 24% reduction in TB incidence rates between 2015 and 2023 [1], globally, the incidence of drug-resistant tuberculosis (DR-TB) remained largely stable between 2020 and 2023 [1] and continues to pose a substantial public health challenge [1], including in sub-Saharan Africa [2, 3]. Most DR-TB cases arise from a mixture of lack of adherence to standard treatments [4]. According to the WHO, South Africa was one of the countries that accounted for about 75% of the global gap between the estimated global number of individuals who developed DR-TB in 2023 and the global number of individuals enrolled on treatment in 2023 [1].

DR-TB treatment has significant patient-incurred costs that present a growing financial burden for patients in countries and communities. Patients in South Africa incur significant costs when accessing diagnosis and treatment for drug-resistant tuberculosis, despite free healthcare [5]. In this regard, within the developed and developing world, recent increases in DR-TB have primarily been associated with smoking [6, 7]. Smoking significantly heightens the risk of developing tuberculosis (TB) and increases the chances of mortality from the disease. This is attributable to the diverse pathophysiological alterations smoking induces in the entire respiratory tract, encompassing local anatomical damage and complex immunological effects [8]. Such deleterious changes then affect the natural lung defense mechanisms, including alterations in cellular and humoral immunity against mycobacterium like TB [9]. It is for this reason that many TB cases worldwide are attributable to five risk factors, undernutrition, HIV infection, alcohol use disorder, diabetes, and smoking [1]. Moreover, there is ample evidence to suggest that smoking influences the clinical manifestations of DR-TB [10]. In this regard, smoking has been indicated as a risk factor that adversely affect the clinical outcomes of patients with DR-TB [11, 12]. Smoking has not only been shown to reduce the effectiveness of drugs in patients with TB and some studies suggest that smoking might even contribute to the development of DR-TB [6, 12].

The importance, and relative cost-effectiveness, of behavioral or lifestyle interventions, such as smoking cessation, make them ideal public health strategies for prevention and management of DR-TB. Without essential contextual information, such as specific risk behaviours, it is unlikely that generalised smoking intervention approaches for patients with DR-TB would succeed in specific populations. This is because such information demonstrates not only the importance of TB for the society, but also the behaviours that drive TB development and severity. Such information is then essential for health-related decision-making in not only assessing the burden of TB, but also for defining priorities for interventions, guideline development, estimation of costs, and further research [13]. Such information is also essential for evaluating the impact of interventions as they demonstrate changes and trends over time [13]. Thus, the aim of this study was to assess the smoking behaviours, cessation attempts, and associated factors among patients with DR-TB.

## Methods

### Design

This cross-sectional study was conducted to collect data between September and December 2016 from all three specialized DR-TB public hospitals located in the eThekwini health district, KwaZulu-Natal (KZN) province, South Africa. These three specialized hospitals serve a population of 11.5 million individuals with an average annual income of 29,400 South African Rands (ZAR) or 1,247 Pound Sterling (£) in an area of around 92,100 square kilometres (km^2^) which is roughly the size of Portugal.

### Participants

Purposive random sampling was applied as proportional to size (i.e. facility [patients]). Eligible participants had to be aged 18 years or older, currently undergoing DR-TB treatment, and self-report as smokers. A sample size of 196 participants was calculated with the assistance of an independent biostatistician using the formula, n = Z2(1-α/2)pq/d2 (where Z(1-α/2) = 1.96 at 95% confidence; p = proportion of reporting a particular barrier for smoking cessation, q = 1-p; d = absolute allowable error incorporating statistical variables) using a finite population size of 400 patients.

### Data collection

A structured interviewer-administered questionnaire was adapted from the existing published Global Adult Tobacco Survey, and has previously been demonstrated to be a reliable tool [14]. Questions were included to determine participant characteristics and smoking behavior, including quantity of smoking, smoking initiation, smoking cessation, age at smoking initiation, and sources of information about the risks of smoking [15]. Concerning the socioeconomic status of the participants and lack of technology and infrastructure, the survey was administered using an anonymous paper questionnaire both in English and the local IsiZulu language by trained research assistants. Questionnaires were completed away from the presence of attending healthcare staff to minimise the potential bias in responses. Ethical approval was obtained from the institutional review boards of the University of KwaZulu-Natal (BE 493/14). Institutional approval was also obtained from the hospital management of each study site. All participants signed the informed consent following an explanation of the purpose of the study as well as their rights as research participants. Prospective participants for the study were recruited through hospital records to identify eligible individuals from admissions and patient databases at the specialised DR-TB hospitals in KwaZulu-Natal. Direct contact was made with patients during their regular visits or treatment sessions at these hospitals. Recruitment materials, such as posters and flyers, were also strategically placed in the hospital waiting areas frequented by DR-TB patients, and word-of-mouth through current participants or healthcare workers further informed potential candidates about the study.

### Data analysis

Statistical analyses were performed by IBM Statistical Package for the Social Sciences (SPSS) ver. 26.0 (IBM Co., Armonk, NY, USA). Descriptive statistics were generated for the study and data were summarised using descriptive statistics such as means, standard deviations, and the range for the numeric data such as age of participants, whilst the frequency, and percentages were used for the categorical data, including sex, and race. These were displayed in frequency tables.

The study involved collecting various types of data, which included demographic, behavioural, and contextual information about the participants. Demographic data, such as age, sex, educational level, and employment status, provided insights into the socioeconomic and personal characteristics of the participants. Behavioural data focused on smoking habits, including the frequency and duration of smoking, timing of the first cigarette of the day, types of cigarettes used, and attempts to quit smoking. Contextual data encompassed factors influencing smoking cessation, such as advice from healthcare providers, the use of support mechanisms like counselling or nicotine replacement therapy, and sources of information about the risks of smoking.

Handling these types of data required a structured approach to ensure accuracy, confidentiality, and usability. A structured interviewer-administered questionnaire adapted from the Global Adult Tobacco Survey was used to collect data, ensuring consistency and reliability. The questionnaires were administered anonymously in both English and the local IsiZulu language by trained research assistants, minimising bias and enhancing participant comfort. Data collection took place away from attending healthcare staff to ensure honest responses. Ethical considerations were prioritised, with all participants providing written informed consent after being briefed about the study’s purpose and their rights. Participant anonymity was safeguarded throughout, in compliance with ethical standards such as the South African Protection of Personal Information Act (POPIA).

Before importing the collected information into the statistical software package, the data was captured through a series of organised steps to ensure accuracy and consistency. Data collection began with structured, interviewer-administered paper questionnaires, where research assistants recorded responses in real-time. Once completed, the responses were reviewed and coded, with categorical data such as sex, smoking habits, and educational levels assigned numerical codes for easier entry and analysis (e.g., 1 = Male, 2 = Female). A data entry template, created in Microsoft Excel, was prepared with predefined columns for each variable to maintain uniformity in data organisation. The primary author manually inputted the coded responses into the template. Validation checks were implemented during the entry process to identify and correct discrepancies, such as setting allowable ranges for numerical responses or ensuring no fields were left blank. Following this, the dataset underwent a cleaning process to address missing values, resolve inconsistencies, and verify accuracy against the original paper questionnaires. Once finalised, the data was formatted for compatibility with the statistical software, such as saving it as a.csv file for import into IBM SPSS.

## Results

### Participants characteristics

One hundred and ninety-six (196) participants responded to the structured interviewer-administered questionnaire adapted from the Global Adult Tobacco Survey. Table 1 presents the characteristics of the study participants. The mean age of the participants was 36.13 ± 10.27 years, with 172 (88.6%) identifying as male and 24 (11.4%) as female. Among the participants, 136 (64.5%) had completed secondary school, while 45 (21.3%) had completed primary school. In terms of employment status, 124 (63.2%) were unemployed, 60 (30.6%) were employed, 5 (2.5%) were full-time students, and one participant was retired.

**Table 1:** Characteristics of patients with drug-resistant tuberculosis (DR-TB) in South Africa (*n* = 196)

Variables	Categories	Results (*n*/%)
Gender	Male	172/87.8
	Female	24/12.2
Race	Black	188/95.9
	Mixed descent	3/1.5
	White	1/0.5
	Indian	4/2.0
Age	18–20	9/4.6
	21–30	61/31.1
	31–40	64/32.7
	41–50	48/24.5
	51 and above	14/7.1
Educational qualification	No formal schooling	14/7.1
	Primary school completed	41/21.0
	Secondary school completed	131/66.8
	Tertiary school completed	9/4.6
	No answer	1/0.5
Employment Status	Employed	60/30.6
	Unemployed	124/63.2
	Student	5/2.6
	Self-employed	6/3.1
	Retired	
	1/0.5	

### Smoking behavior

Tables 2 and 3 provide an overview of the smoking behavior among the study participants. All participants self-reported as smokers, with 95.3% reporting smoking cigarettes daily. Among these participants, 65% had been smoking daily for at least the past three months. The mean age for the first smoking experience was reported as 14.89 years ± 7.94 SD, with 96 (45.5%) of participants trying smoking before the age of 18. Notably, three participants reported starting smoking at the young age of 10 years. Regarding the timing of the first cigarette of the day, 71 (36.2%) of participants smoked within five minutes of waking up, 50 (25.5) within 30 min, and 61 (31.1) within one hour.

**Table 2:** Smoking behavior of patients with drug-resistant tuberculosis (DR-TB) in South Africa (*n* = 196)

Variables	Response categories	(*n*/%)
Smoke cigarettes daily	Yes	187/95.4
	No	7/3.6
	Don’t know	1/0.5
	No answer	1/0.5
Smoking patterns in the past three months	Daily	136/69.3
	Less than daily	15/7.7
	Not at all	36/18.4
	No answer	9/4.6
Age categories of first-time smoking	10–17 years	91/46.4
	18–24 years	61/31.1
	25–32 years	11/5.6
	33–40	1/0.5
	41 and above	1/0.5
	No answer	31/15.8
First smoke of the day (From awakening)	Within 5 min	71/36.2
	6–30 min	50/25.5
	31–60 min	61/31.1
	More than 60 min	2/1.0
	No response	12/6.1

Table 3 summarizes the average number of cigarettes smoked daily and weekly by patients. On average, participants smoked 6.73 ± 6.27 manufactured cigarettes per day, totaling 47.08 ± 43.82 per week. For hand-rolled cigarettes, the daily average was 0.86 ± 2.71, amounting to 5.80 ± 18.74 per week. Additionally, other types of cigarettes were consumed at a lower rate, averaging 0.46 ± 1.63 per day and 3.23 ± 11.43 per week. These findings highlight the predominant use of manufactured cigarettes among the participants.

**Table 3:** Number of cigarettes smoked per day and per week in patients with drug-resistant tuberculosis (DR-TB) in South Africa

Manufactured cigarettes (per day)	Manufactured cigarettes (per day)	Hand-rolled cigarettes (per day)	Hand-rolled cigarettes (per day)	Other (per day)	Other (per week)
6.73 ± 6.27	47.08 ± 43.82	0.86 ± 2.71	5.80 ± 18.74	0.46 ± 1.63	3.23 ± 11.43

Data reported as means ± standard deviation

## Discussion

Despite the WHO African region achieving a 24% reduction in TB incidence rates between 2015 and 2023 [1], the global burden of DR-TB remained relatively stable from 2020 to 2023 [1]. DR-TB continues to present a significant public health challenge [1], particularly in sub-Saharan Africa [2, 3], with South Africa among the 10 countries accounting for approximately 75% of the global gap between estimated DR-TB cases and those enrolled in treatment in 2023 [1]. As such, there is a need for reliable data on TB risk behaviors for health-related decision-making, defining priorities for interventions, guideline development, estimation of costs, and for evaluating the impact of interventions as they demonstrate changes and trends over time [13]. This may provide an explanation as to why some countries are battling to provide effective preventative and treatment strategies and policies to combat DR-TB and their prominent risk factors, such as smoking [6]. Such information is important in that it may be that the association between smoking and DR-TB differs in specific contexts or countries. This is because lifestyle interventions, such as smoking cessation interventions, may present be cost-effective and effective at reducing DR-TB development, morbidity, and mortality [16].

Studies have explored the characteristics and smoking behaviors of patients with TB [17, 18], yet there remains a paucity of contextual data specifically focusing on those DR-TB. Such information is crucial, as evidence suggests notable differences between TB and DR-TB regarding smoking prevalence, socio-economic factors, and treatment history, among other variables. For instance, a study involving 240 Indian patients with DR-TB reported that 66% were male, and 46% belonged to a poor socio-economic background [19]. Similarly, findings from a Brazilian study [20], revealed that most DR-TB patients were working-age males with less than eight years of education. However, contrasting insights emerged from research in Portugal, where smoking habits were not identified as a significant threat to tuberculosis control among a sample of 119 DR-TB patients [21]. These variations underscore the importance of contextualized data in understanding and addressing the nuances of DR-TB.

In line with the aim of the study to determine the characteristics among patients with DR-TB in South Africa, the finding of this study and previous research [20] that almost a quarter of participants with DR-TB had only completed primary school should be a concern to policy makers and healthcare professionals involved in the prevention and management of DR-TB. This is because an increased mortality from TB, and other infectious diseases, has been found among less educated individuals [22]. These findings suggest that when planning and implementing TB and DR-TB prevention and control programs, greater attention should be paid to the less educated population at highest risk of this disease [22].

Specifically, the findings of this study suggest that DR-TB control in South Africa, might benefit from general interventions aimed at reducing tobacco use in middle childhood (9–12 years old) given that this study found a mean age for the first smoking experience of 14.89 years old. Given that behavioral risk factors are often adopted earlier in life, this information provides a window period for behavior modification for the prevention of DR-TB. Although no causal links yet exist on the number of cigarettes smoked daily and DR-TB, it is plausible that an increased daily smoking volume could have an association with poorer prognosis and outcomes. As such, there may be a need for interventions and guidelines for smokers with TB to also focus on smoking reduction as well as cessation. Further, it has also been proposed that smoking reduction promotes rather than deters cessation as smoking reduction may be a more attainable goal compared to complete cessation and once achieved, smoking reduction may encourage further efforts to achieve cessation [23]. An interesting finding of this study is that most of the sampled smokers smoked manufactured cigarettes while less than a quarter smoked self-rolled cigarettes from loose tobacco. This contrasts with the supposition that “roll your own” cigarettes, tend to be at most equally dangerous, but more addictive than manufactured cigarettes [24]. What is particularly concerning is that in this study, only 64% of participants disclosed their smoking behavior on admission to the specialized DR-TB public hospitals. While the reasons for this are unknown, such lack of disclosure could bypass necessary TB and DR-TB prevention and control programs aimed at smoking as an independent risk factor for prognosis and outcomes of DR-TB.

Previously, smoking has demonstrated to increase TB mortality nine-fold [25]. However, when patients with TB quit smoking, their risk of mortality is reduced by more than half to a level not different from non-smokers [25]. This reinforces the importance of smoking cessation interventions in TB control. This study demonstrates that smoking cessation interventions may provide a valuable, cost-effective, and wanted intervention in that most of the participants indicated that they attempted to quit smoking in the past 12 months. Interestingly, cigarette costs were one of the reasons for attempted smoking cessation among 8.1% of participants. This indicates that the progressive tax on cigarette products plays an important role in tobacco control. Problematically, there is no clear message from health professionals or the specialized hospitals to quit. This study also demonstrated that there is a need to develop smoking cessation interventions that stress the urgency of quitting smoking since half of the participants reported an interest to quit smoking at some time in the future.

In this study, the high prevalence of cigarette smoking cannot be linked to a lack of awareness of the health risks of smoking cigarette. This is because participants reported being aware of the health risks of smoking cigarettes arising from several sources of information including, newspapers, television, radio, billboards, hospital signage, and health warnings on cigarette packaging, with cigarette packaging being the most reported.

The study has several limitations that should be considered when interpreting its findings. The cross-sectional design of the study limits the ability to establish causal relationships between smoking behaviors and health outcomes among DR-TB patients. The self-reported data on smoking behaviors may be subject to recall bias or underreporting, particularly given the low disclosure rate of smoking behavior upon hospital admission. Additionally, the study was conducted in three specialized hospitals in KwaZulu-Natal, which may limit the generalizability of the findings to other regions or settings. Another limitation is the reliance on structured interviewer-administered questionnaires, which may have introduced response bias due to social desirability, especially in the presence of sensitive questions about smoking. The study also did not account for potential confounding variables such as co-morbidities or substance use that could influence smoking behaviors and cessation outcomes.

## Conclusions

Based on the study’s finding that more than half of the participants expressed intentions to quit smoking, it is essential to recognize the importance of building on this intention through targeted interventions that provide both psychological and practical support for those who are ready to quit. In this regard, programs that combine behavioral therapy with pharmacological treatments, such as nicotine replacement therapy or medications, could be emphasized as effective methods to enhance success rates. Continued efforts should focus on increasing awareness about the health risks of smoking and the benefits of quitting, while also addressing potential barriers such as stress, triggers, and social influences. Tailored cessation programs that cater to specific populations (e.g., age, socio-economic status, or previous smoking history) could also improve outcomes by addressing unique needs. By ensuring that participants have access to ongoing support and resources, we can help transform their intention into long-term success.

## Data Availability

Raw data for datasets are not publicly available to safeguard participan privacy, especially those from vulnerable populations (Sect. 11 of the South African Protection of Personal Information Act (POPIA)). The anonymised data supporting this research are available from the authors.

## Abbreviations

DR-TB: Drug-resistant tuberculosis
HIV: Human Immunodeficiency Virus
KZN: KwaZulu-Natal
SD: Standard deviation
SPSS: Statistical Package for the Social Sciences
TB: Mycobacterium tuberculosis
WHO: World Health Organization
